# Uncovering key molecules and immune landscape in cholestatic liver injury: implications for pathogenesis and drug therapy

**DOI:** 10.3389/fphar.2023.1171512

**Published:** 2023-05-09

**Authors:** Shuailing Song, Xiao Li, Chong Geng, Yaoyu Guo, Yi Yang, Chunhui Wang

**Affiliations:** ^1^ Department of Gastroenterology, West China Hospital, Sichuan University, Chengdu, China; ^2^ Laboratory of Gastroenterology and Hepatology, West China Hospital, Sichuan University, Chengdu, China; ^3^ Department of Laboratory Medicine, Sichuan Provincial People’s Hospital, University of Electronic Science and Technology of China, Chengdu, China

**Keywords:** miRNA-mRNA network, cholestatic liver injury, immune infiltration, monocyte, SYK, UDCA

## Abstract

**Background:** Cholestasis is a common pathological process in a variety of liver diseases that may lead to liver fibrosis, cirrhosis, and even liver failure. Cholestasis relief has been regarded as a principal target in the management of multiple chronic cholestasis liver diseases like primary sclerosing cholangitis (PSC) and primary biliary cholangitis (PBC) at present. However, complicated pathogenesis and limited acknowledgments fettered therapeutic development. Therefore, this study aimed to systematically analyze miRNA-mRNA regulatory networks in cholestatic liver injury in order to provide new treatment strategies.

**Methods:** Gene Expression Omnibus (GEO) database (GSE159676) was used to screen differentially expressed hepatic miRNAs and mRNAs in the PSC *vs.* control comparison and the PBC *vs.* control comparison, respectively. MiRWalk 2.0 tool was used to predict miRNA-mRNA pairs. Subsequently, functional analysis and immune cell infiltration analysis were performed to explore the pivotal functions of the target genes. RT-PCR was used to verify the result.

**Results:** In total, a miRNA-mRNA network including 6 miRNAs (miR-122, miR-30e, let-7c, miR-107, miR-503, and miR-192) and 8 hub genes (PTPRC, TYROBP, LCP2, RAC2, SYK, TLR2, CD53, and LAPTM5) was constructed in cholestasis. Functional analysis revealed that these genes were mainly involved in the regulation of the immune system. Further analysis revealed that resting memory CD4 T cells and monocytes could potentially participate in cholestatic liver injury. The expressions of DEMis and eight hub genes were verified in ANIT-induced and BDL-induced cholestatic mouse models. Furthermore, SYK was found to have an impact on the response to UDCA, and its mechanism was possibly associated with complement activation and monocyte reduction.

**Conclusion:** In the present study, a miRNA-mRNA regulatory network was constructed in cholestatic liver injury, which mostly mediated immune-related pathways. Moreover, the targeted gene SYK and monocytes were found to be related to UDCA response in PBC.

## Introduction

Cholestasis is a basic pathological change shared by various liver disorders, causes of which include obstruction, genetics, pregnancy, and drug induction. Most chronic cholestatic liver diseases like primary biliary cholangitis (PBC) and primary sclerosing cholangitis (PSC), usually present a spectrum of disease severity - from no or slight symptoms to cholestasis to biliary cirrhosis resulting in end-stage liver disease due to their cumulative nature (Karlsen et al., 2017; Galoosian et al., 2020). On the one hand, it is easily found that cholestasis is implicated in disease progression. Unsolved disturbances of bile acid metabolism could over-activate a series of inflammation and immune responses (Santiago et al., 2018; Woolbright and Jaeschke, 2019). On the other hand, long-term cholestasis could cause irreversible destruction of hepatocytes and bile ducts. Thus, quick and effective release of cholestasis is always a principal target for chronic cholestatic liver diseases. Ursodeoxycholic acid (UDCA) therapy is the most established drug therapy for cholestasis (Beuers et al., 2015). It improves impaired bile formation and flow mainly through antiapoptotic, anti-inflammatory, and immunomodulatory effects. Currently, UDCA treatment may slow the progression of chronic cholangiopathies, but has limited or no proven efficacy in various chronic cholestatic liver injuries and cannot heal them (Beuers et al., 2015). The nuclear receptors farnesoid X receptor (FXR), peroxisome proliferator-activated receptor α (PPARα), and pregnane X receptor (PXR) are modifiers of bile formation and, at present, are under investigation as promising targets for therapeutic interventions in cholestasis. However, their efficacy and safety still need to be supported by more real-world data (Bachs et al., 1992; Corpechot et al., 2018; Floreani et al., 2022). Thus, these considerations emphasized the need for deeper mechanism explorations of cholestatic liver injury in order to provide novel therapeutic targets.

MicroRNAs (miRNAs) are a burgeoning research direction in cholestatic liver diseases. Increasing pieces of evidence have revealed the principal roles of miRNAs in the development of cholestatic liver diseases (Marin et al., 2014; Huang et al., 2020). It was reported that miRNAs shared a link with immune dysregulation in cholestatic liver injury, including effector CD8^+^ T cell upregulation (Ando et al., 2013) and Th17 cell redistribution (Song et al., 2018). Therefore, a comprehensive analysis of the miRNA-mRNA regulatory network in cholestatic liver injury would provide deeper insight into the mechanism as well as potential therapeutic targets.

In the present study, we utilized data from two cholestatic liver diseases-PSC and PBC to construct a miRNA-mRNA regulatory network in order to identify pivotal modulators in cholestatic liver injury. The proposed hypothesis is that differentially expressed miRNAs (DEMis) and targeted mRNAs participated in cholestatic liver injury partly through modulating the immune system, represented by resting memory CD4 T cells and monocytes. Among them, aberrant SYK expression and monocyte infiltration were also related to high-risk PBC, which presented an insufficient response to UDCA.

## Materials and methods

### Data sources

The microarray datasets were downloaded from the GEO database. The gene expression profile dataset GSE159676 was performed on Platform GPL6244, with liver tissues from 12 PSC patients, 3 PBC patients, and 5 healthy people. In order to uncover genes related to cholestatic liver injury, differential genes were screened according to the PSC or PBC groups and the control group.

Dataset GSE166867 contained liver tissue from shame operation mice (n = 5) and 21-day bile duct ligation mice (n = 5) was used to verify the identified hub genes.

Dataset GSE79850 performed on GPL19965 contained the first liver biopsies from 16 PBC patients. According to the results from a minimum of 15 years of follow-up, 7 cases responding fully to UDCA according to Paris 1 criteria were defined as a low-risk group, and the other 9 cases who were nonresponsive to UDCA after 1-year treatment and requiring further liver transplantation were defined as a high-risk group. Gene expressions between the high-risk group and the low-risk group were compared and analyzed to excavate potential factors associated with UDCA response.

### Data preprocessing and construction of miRNA-miRNA regulatory network

Differentially expressed genes (DEGs) in the PSC *vs.* control comparison and the PBC *vs.* control comparison in GSE159676 were obtained using GEO2R. Because of the small sample size, DEMis were screened out by an adjusted *p*-value < 0.05 and fold change (FC) > 1.2, and the intersection between the two comparisons was selected as common DEMis in cholestasis. Then, the overlapping differentially expressed mRNAs (DEMs) screened out by an adjusted *p*-value < 0.05 were chosen to match with DEMis by the miRWalk V2.0 database. To show the expression of DEMis and DEMs in different groups, volcano maps, Venn diagrams, and heatmaps were drawn by Hiplot (https://hiplot.com.cn), an online bioinformatics data visualization platform. And a miRNA-mRNA regulatory network was visualized by Cytoscape software (Smoot et al., 2011).

### Gene ontology (GO) annotation and kyoto encyclopedia of genes and genomes (KEGG) pathway enrichment analysis

After building a cholestasis-associated miRNA-mRNA regulatory network in GSE159676, Gene Ontology (GO) analysis was then performed on the targeted mRNAs by ClueGo plugin of Cytoscape (Bindea et al., 2009), which revealed the functional enrichment including biological processes (BPs), cellular components (CCs), and molecular functions (MFs). In addition, Kyoto Encyclopedia of Genes and Genomes (KEGG) pathways enrichment analysis was also carried out and visualized by Hiplot. *p* < 0.05 was considered statistically significant.

### Construction of protein-protein interaction (PPI) network and identification of hub genes

To understand the interactions among the target genes, DEMs were also uploaded to STRING (http://string-db.org) database to construct a Protein-Protein Interaction (PPI) network with confidence scores ≥ 0.4. Then, cluster analysis was performed utilizing MCODE plugin of Cytoscape, and clusters with the top 2 scores were visualized by Cytoscape. Additionally, the targeted genes were also ranked by degree scores using CytoHubba plugin. Hub genes, which were defined as genes that played an essential role in the network, were determined by a combination of MCODE algorithm and CytoHubba degree method (Chin et al., 2014). A miRNA-hub gene regulatory network was then constructed, and KEGG pathway analysis was further performed by the clusterProfiler package.

### Immune cell infiltration analysis

To determine the immune landscape of samples in GSE159776 and GSE79850, immune cell infiltration analysis was performed by the CIBERSORT package. The proportions of different immune cells in each sample were visualized by the ggplot2 package.

### Animal model

Wild-type C57BL/6 mice (6–8 weeks, 14–18 g) were purchased from Chengdu Dashuo Biotechnology Co., Ltd. (Sichuan, China). α-Naphthyl isothiocyanate (ANIT) was obtained from Macklin (Shanghai, China). For ANIT-induced cholestasis model, mice were randomly assigned to the control group and the cholestatic liver injury group (ANIT group). The ANIT group mice were gavaged with ANIT dissolved in olive oil at a dose of 50 mg/kg, and the control group was given a consistent volume of olive oil. All mice were sacrificed 48 h after treatment. For BDL-induced cholestasis model, mice were randomly divided into sham group and BDL group. Mice were anesthetized by breathing isoflurane (1.5 vol%). BDL or sham procedure was performed after midline laparotomy. All mice were sacrificed 21 days after BDL. Serum and liver samples were harvested and preserved in liquid nitrogen immediately. All animal study procedures were performed in accordance with the approved guidelines, and all experimental protocols were approved by the Ethical Committee of the West China Hospital of Sichuan University.

### Hematoxylin and eosin (H&E) staining and immunofluorescence staining

Tissue sections (4 μm) were obtained from paraffin-embedded samples. H&E staining and myeloperoxidase (MPO) immunofluorescence staining were performed according to standard protocols in order to evaluate liver inflammation and injury. Specific primary antibiotic for MPO (1:400, Abcam, United States), and secondary antibiotic goat anti-rabbit IgG H&L (1:400, Abcam, United States) were used. Images were obtained by a microscope (Olympus, Japan).

### RNA isolation and RT-qPCR analysis

Total RNA from the liver samples was extracted using a Total RNA kit (Forgene, Sichuan, China) according to the manufacturer’s instructions. A miRNA reverse transcription kit (Vazyme, Jiangsu, China) and a first-strand cDNA synthesis kit (Forgene, Sichuan, China) were applied to synthesize cDNA. Reverse transcriptase quantity PCR (RT-qPCR) was accomplished using SYBR Green PCR Master Mix (Forgene, Sichuan, China). Primers for RT-qPCR are presented in Supplementary Table S1. The same reverse primer with the sequence 5′-AGT​GCA​GGG​TCC​GAG​GTA​TT-3′ was used for all miRNAs. The average expression levels of liver miRNAs and mRNAs were normalized to U6 and GAPDH, respectively. Relative gene expression was determined by the 2^−ΔΔCt^ method.

### Gene Set Enrichment Analysis (GSEA) and gene set variation analysis (GSVA)

In order to obtain the differential BPs between different groups in GSE79850, Gene Set Enrichment Analysis (GSEA) was further performed using the fgsea package. In addition, GSVA was used to determine the significantly altered pathways via GSVA package. Results were presented through gseaplots. *p*-value < 0.05 was considered statistically significant.

### Identification of small-molecule compounds

The Connectivity Map (CMap) (Subramanian et al., 2017) (https://clue.io) is an open resource that links genes, compounds, and diseases by similar or opposite gene signatures. DEMs between the high-risk group and low-risk group in GSE79850 were screened out by a *p*-value <0.05. The top 100 significant upregulated DEMs and all downregulated DEMs (68 in total) were uploaded into the upregulated list and downregulated list of the CMap database, respectively, to predict small molecules associated with UDCA response. Compounds that could reverse the altered expression of DEMs were considered potential therapeutic agents for high-risk PBC. False discovery rate (FDR) < 0.05 was set as the screening criteria. The top 50 compounds and their mechanisms of action were visualized by the ggplot2 package.

### Statistical analysis

All data are presented as the mean ± standard error (SEM). Student’s t-test was carried out to compare two groups, and one-way analysis of variance (ANOVA) was applied for multigroup (≥3) comparisons by SPSS 16.0 (Chicago, United States). The bar charts and the ROC curve analysis were completed by GraphPad Prism 8.0 (California, United States). *p* < 0.05 was considered statistically significant.

## Results

### Construction of a miRNA-mRNA regulatory network

**FIGURE 1:**
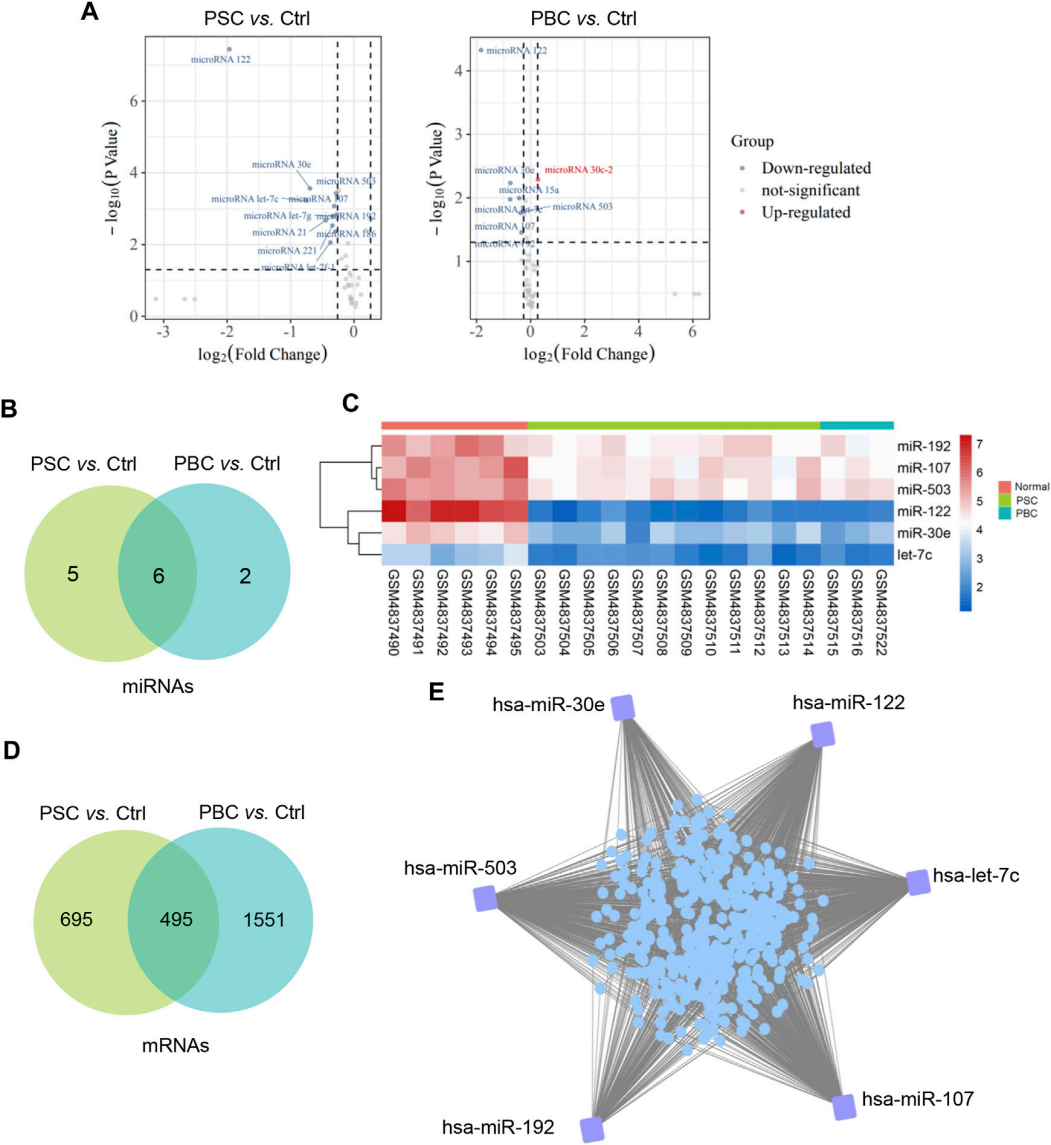
Construction of a miRNA-mRNA regulatory network. **(A)**Volcano plot of differentially expressed miRNAs of PSC *vs.* Ctrl and PBC *vs.* Ctrl in GSE159676; **(B)** Common DEMis between PSC *vs.* Ctrl comparison and PBC *vs.* Ctrl comparison; **(C)** A heatmap represents common DEMis; **(D)** Common DEMs between PSC *vs.* Ctrl comparison and PBC *vs.* Ctrl comparison; **(E)** A miRNA-mRNA regulatory network in cholestatic liver injury. PSC, primary sclerosing cholangitis; PBC, primary biliary cholangitis; Ctrl, control; DEMis, differentially expressed miRNAs; DEMs, differentially expressed mRNAs.

According to the filtering criterion described above, 11 downregulated DEMis between the PSC group and the control group were identified. When comparing the PBC group to the control, 8 altered miRNAs including 1 upregulated miRNA and 7 downregulated miRNAs were observed. The distributions of DEMi expression were intuitively illustrated by volcano maps (Figure 1A). Furthermore, the intersection of the candidate DEMis from the two comparisons was visualized by a Venn diagram (Figure 1B) and a heatmap (Figure 1C). The shared DEMis were also listed in Table 1, including 6 downregulated miRNAs in total (miR-122, miR-30e, let-7c, miR-107, miR-503, and miR-192). In addition, a total of 495 common DEMs were obtained by intersecting the PSC *vs.* control comparison and the PBC *vs.* control comparison (Figure 1D). Based on the above analysis, a regulatory network that connected the 6 DEMis and 437 targeted mRNAs was then constructed (Figure 1E).

**TABLE 1:** Differentially-expressed miRNAs.

Symbol	PSC *vs.* Ctrl		PBC *vs.* Ctrl		Up/Down
	LogFC	Adj.P	LogFC	Adj.P	
hsa-miR-122	−1.97	3.58E-08	−1.84	4.73E-05	Down
hsa-miR-30e	−0.69	2.72E-04	−0.75	5.85E-03	Down
hsa-let-7c	−0.75	5.91E-04	−0.76	1.06E-02	Down
hsa-miR-107	−0.31	8.44E-04	−0.38	1.72E-02	Down
hsa-miR-503	−0.28	3.70E-04	−0.26	1.64E-02	Down
hsa-miR-192	−0.27	1.44E-03	−0.34	3.50E-02	Down

### Enrichment analysis

**FIGURE 2:**
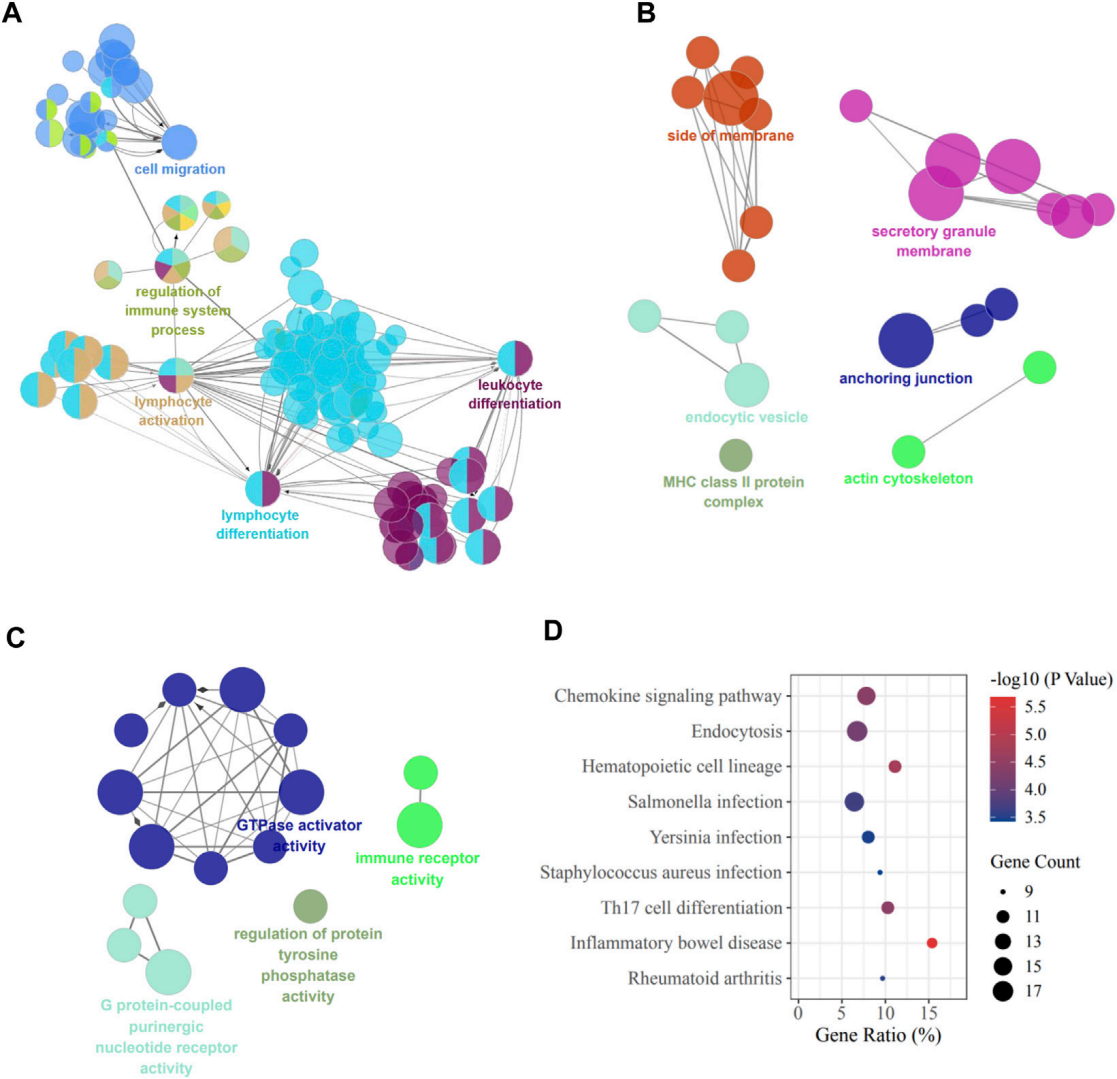
GO and KEGG terms of DEMs. **(A)** Biological process; **(B)** Cellular component; **(C)** Molecular function; **(D)** KEGG pathway enrichment. DEMs, differentially expressed mRNAs.

To investigate the functions of the 437 target genes, GO annotation and KEGG pathway analysis were performed utilizing the Cluego plugin of Cytoscape. The results showed that BP terms were significantly enriched in lymphocyte differentiation and activation and immune system-related function (Figure 2A). The CC terms included side of membrane, secretory granule membrane, anchoring junction, actin cytoskeleton, and MHC class Ⅱ protein complex (Figure 2B). And the most enriched entries for MF terms were activities associated with GTPase, G protein-coupled purinergic nucleotide receptor, protein tyrosine phosphatase, and immune receptor (Figure 2C). In addition, the most significantly enriched pathways of KEGG are shown in Figure 2D, including pathways related to chemokine signaling, Th17 cell differentiation, and various infections, which were critical for the pathological development of cholestatic liver injury.

### Construction of PPI network and identification of hub genes

**FIGURE 3:**
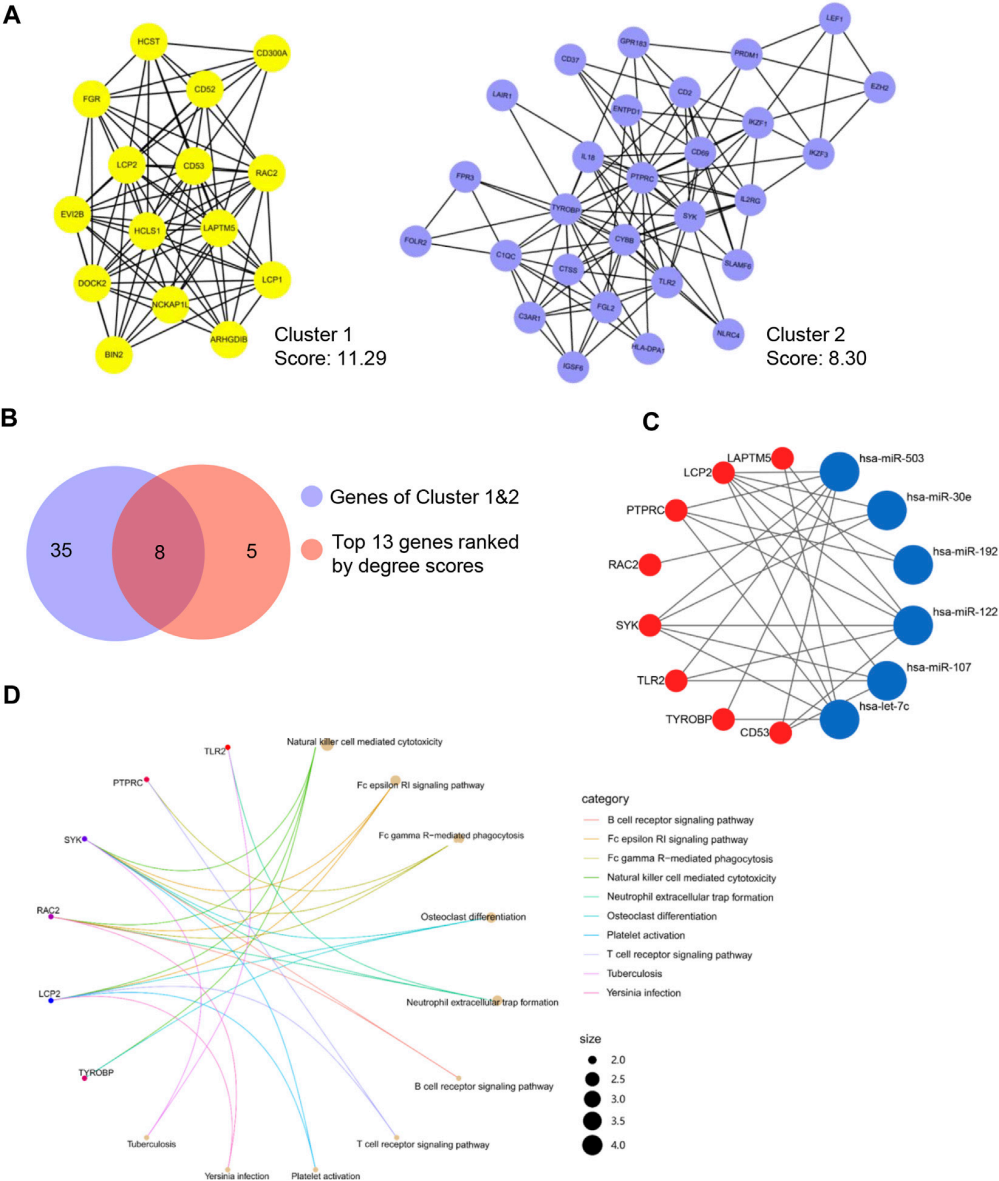
Identification of the hub genes. **(A)** Top 2 clusters identified by MCODE; **(B)** Common hub genes identified by MCODE and cytoHubba; **(C)** A miRNA-hub gene network; **(D)** KEGG pathway enrichment of hub genes.

To illustrate the interactions among the 437 target genes, a PPI network was further constructed using the STRING database. Based on this network data, MCODE plugin was applied to identify gene clusters, and the top 2 clusters ranked by the score were shown in Figure 3A. Besides, the key genes were also ranked by the degree method through CytoHubba plugin (Table 2). In order to obtain the hub genes, these genes in Cluster 1 and Cluster 2 were further filtered by matching the 13 genes with the top 10° scores (Figure 3B). Then, we got 8 hub genes: PTPRC, TYROBP, LCP2, RAC2, SYK, TLR2, CD53, and LAPTM5. A miRNA-hub gene regulatory network was then constructed, with SYK and LCP2 being the targets of most DEMis (Figure 3C). Notably, results from KEGG pathway analysis highlighted impaired immune-related pathways. Pathways like the B cell receptor signaling pathway, Fc epsilon RI signaling pathway, and natural killer (NK) cell-mediated cytotoxicity were significantly enriched (Figure 3D).

**TABLE 2:** Top 13 genes in network ranked by degree method.

Rank	Symbol	Score
1	PTPRC	84
2	TYROBP	62
3	LCP2	51
4	RAC2	45
5	CD44	40
6	CD48	40
7	SYK	39
8	FOXP3	38
9	TLR2	38
10	IL7R	37
11	CXCL8	37
12	CD53	37
13	LAPTM5	37

### Immune cell infiltration analysis

**FIGURE 4:**
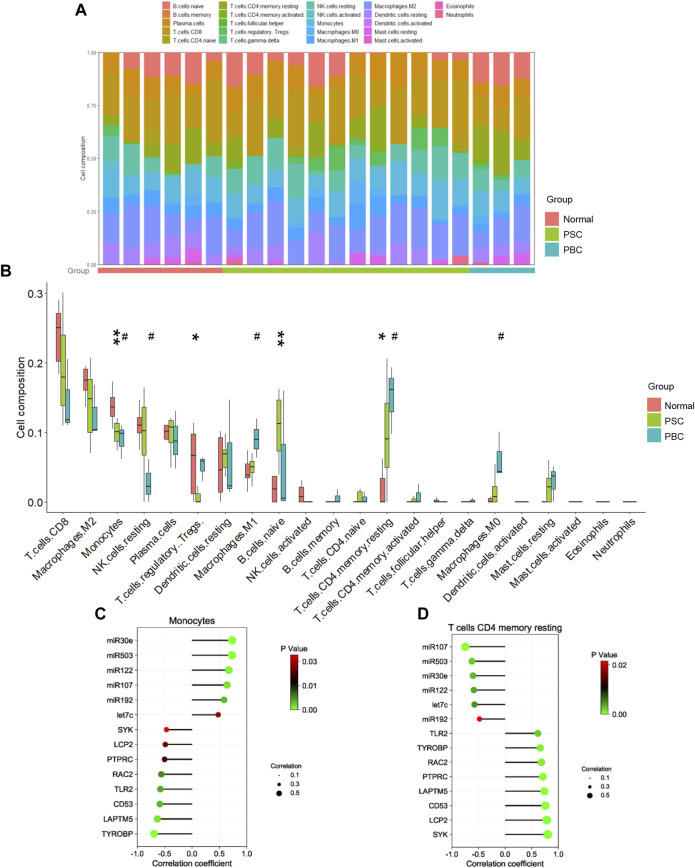
Immune cell analysis in cholestatic liver injury. **(A, B)** Immune cell proportion in PSC, PBC and control livers; **(C)** Correlation between monocyte proportion and DEMis/DEMs; **(D)** Correlation between CD4 memory resting T cell proportion and DEMis/DEMs. * represents comparing the PSC group with the normal group; # represents comparing the PBC group with the normal group. *or # *p* < 0.05, ** *p* < 0.01.

As concluded from the functional analysis, the dysfunctional immune system was closely related to cholestasis. Herein, we further performed an immune cell infiltration analysis to verify this conclusion. The findings confirmed that cholestasis might influence the hepatic immune milieu by demonstrating that the immunological landscape of PSC or PBC livers was significantly different from that of control livers (Figures 4A, B). Specifically, monocytes, regulatory T cells (Tregs), naive B cells, and resting memory CD4 T cells were found significantly different in the PSC group compared to the normal group. Impaired monocytes, resting NK cells, M1 macrophages, resting memory CD4 T cells, and M0 macrophages were seen in the PBC group compared to the normal group. Notably, lower levels of monocyte infiltration and higher levels of resting memory CD4 T cell infiltration were found in the livers of PSC and PBC simultaneously, suggesting that these two immune cells may be crucial in the development of cholestatic liver injury. Furthermore, correlation analysis suggested that either monocyte proportion or resting memory CD4 T cell proportion was significantly correlated with the expression of DEMis and hub genes (Figures 4C, D). This finding strongly supported the idea that the identified DEMis and hub genes could affect monocyte and resting memory CD4 T cell infiltrations in the course of cholestatic liver injury.

### Verification of the identified miRNA-mRNA network in animal cholestatic models

**FIGURE 5:**
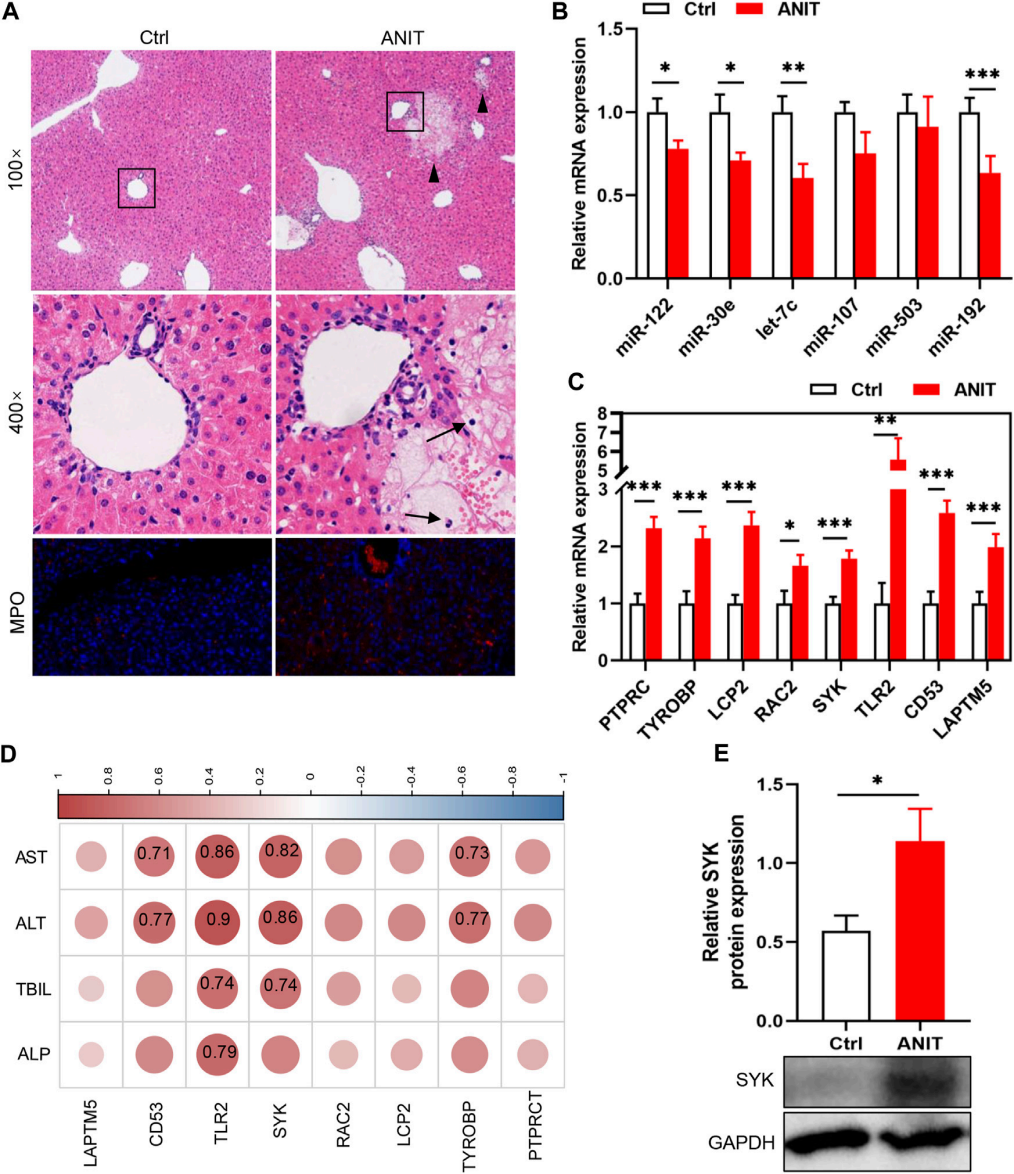
Verification of DEMis/DEMs in ANIT-induced cholestatic mice by RT-qPCR. **(A)** H&E staining and MPO immunofluorescence staining; **(B)** Hepatic DEMi expression; **(C)** Hepatic hub gene expression; **(D)** Correlation between hub genes and biochemical indexes. Numbers represent Pearson’s correlation coefficient (*p* < 0.05); **(E)** SYK protein expression by western blot. Triangles represent liver necrosis; arrows represent immune cells. *p < 0.05, **p < 0.01, ***p < 0.001.

**FIGURE 6:**
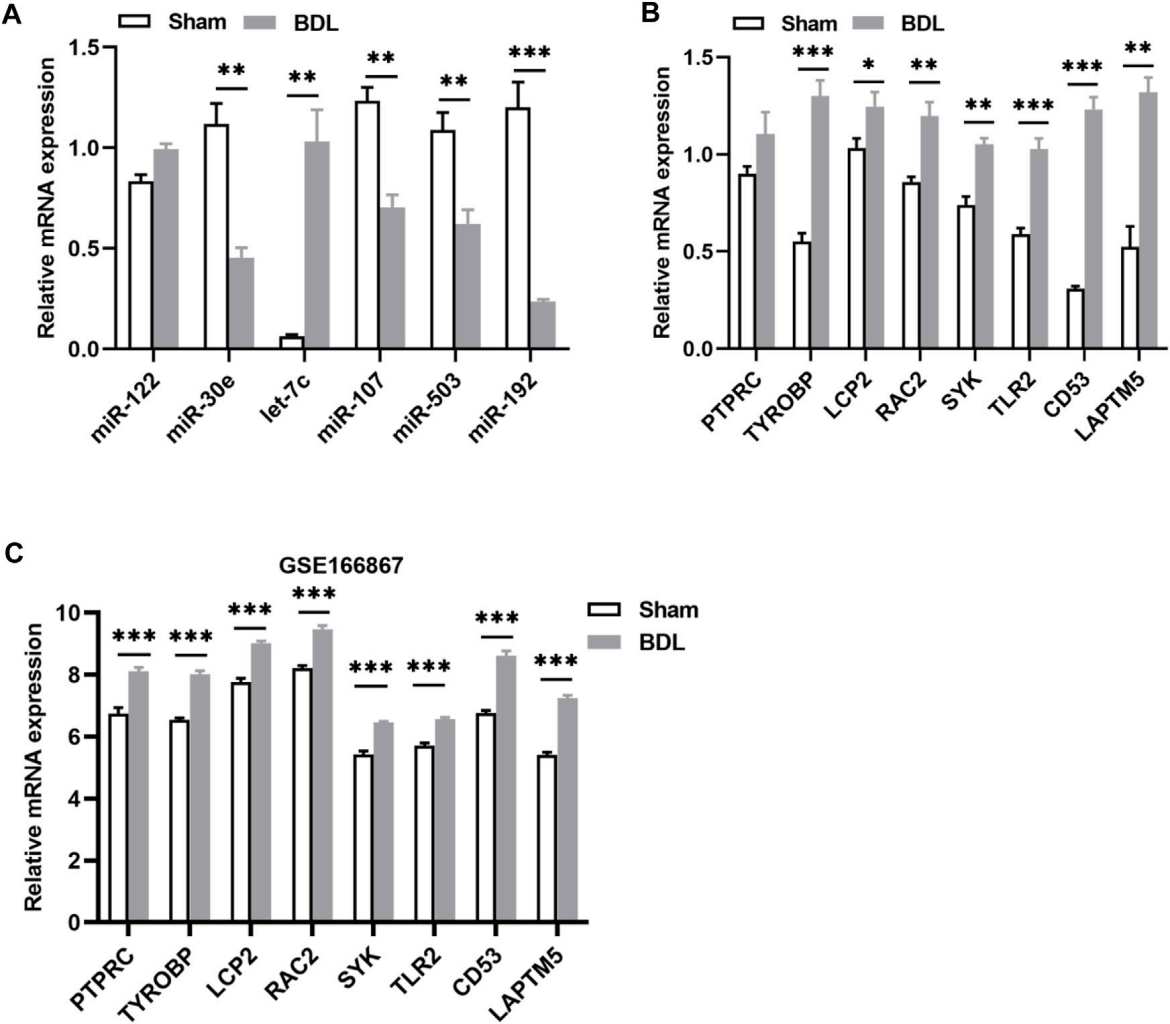
Verification of DEMis/DEMs in BDL-induced cholestatic mice. **(A)** Hepatic DEMi expression by RT-qPCR; **(B)** Hepatic hub gene expression by RT-qPCR; **(C)** Hepatic hub gene expression in GSE166867. *p < 0.05, **p < 0.01, ***p < 0.001.

Two animal models were constructed to verify the results of the bioinformatics analysis. We first detected the levels of the identified DEMis in ANIT-induced cholestatic liver injury mice. As shown in Figure 5A, ANIT treatment induced obvious liver necrosis and immune cell infiltration in the liver, and enhanced MPO staining was also detected in the ANIT group, suggesting that a cholestatic model had been established successfully. In addition, serum biochemical detection also confirmed the result (Supplementary Figure S1). RT-qPCR showed that the expression of four DEMis (miR-122, miR-30e, let-7c, and miR-192) was significantly lower in the ANIT group than in the control group, except for miR-107 and miR-503, which did not reach a significant difference despite a trend toward downregulation in the ANIT group (Figure 5B). We further analyzed the expression of the hub genes in the ANIT-treated and control mice. Consistently, ANIT-induced cholestasis significantly stimulated the hepatic expression of the hub genes (Figure 5C). In order to understand whether the identified miRNA-mRNA regulatory network was involved in the severity of the cholestatic liver injury, we further performed a correlation analysis between the expressions of hub genes and levels of serum biochemical indexes. Results showed that the expressions of SYK and TLR2 were the most significantly correlated (Figure 5D). We suspected that TLR2 and SYK may be closely associated with the severity of the cholestatic liver injury. In addition, elevated SYK protein expression by ANIT treatment was also detected by western blot (Figure 5E). Furthermore, to better mimic the long-term cholestatic-like phenotype of PSC or PBC, we also established a 21-day BDL mouse model. The successful induction of cholestasis by BDL was demonstrated by elevated serum biochemical indices (Supplementary Figure S2). Results showed miR-30e, miR-107, miR-503, and miR-192 were remarkably downregulated in BDL mice (Figure 6A). Furthermore, hub genes except PTPRC were significantly increased after BDL (Figure 6B). We also used an external dataset GSE166867, which contained liver tissues from 21-day BDL mice. As expected, result showed that BDL stimulated the expression of all hub genes (Figure 6C). Taken together, it was demonstrated that a similar miRNA-mRNA network as what we found in PSC and PBC patients was sustained in both animal cholestasis models. Therefore, we believe that the identified miRNA-mRNA regulatory network could help further elucidate the mechanism of cholestasis.

### Molecules involved in the UDCA response

**FIGURE 7:**
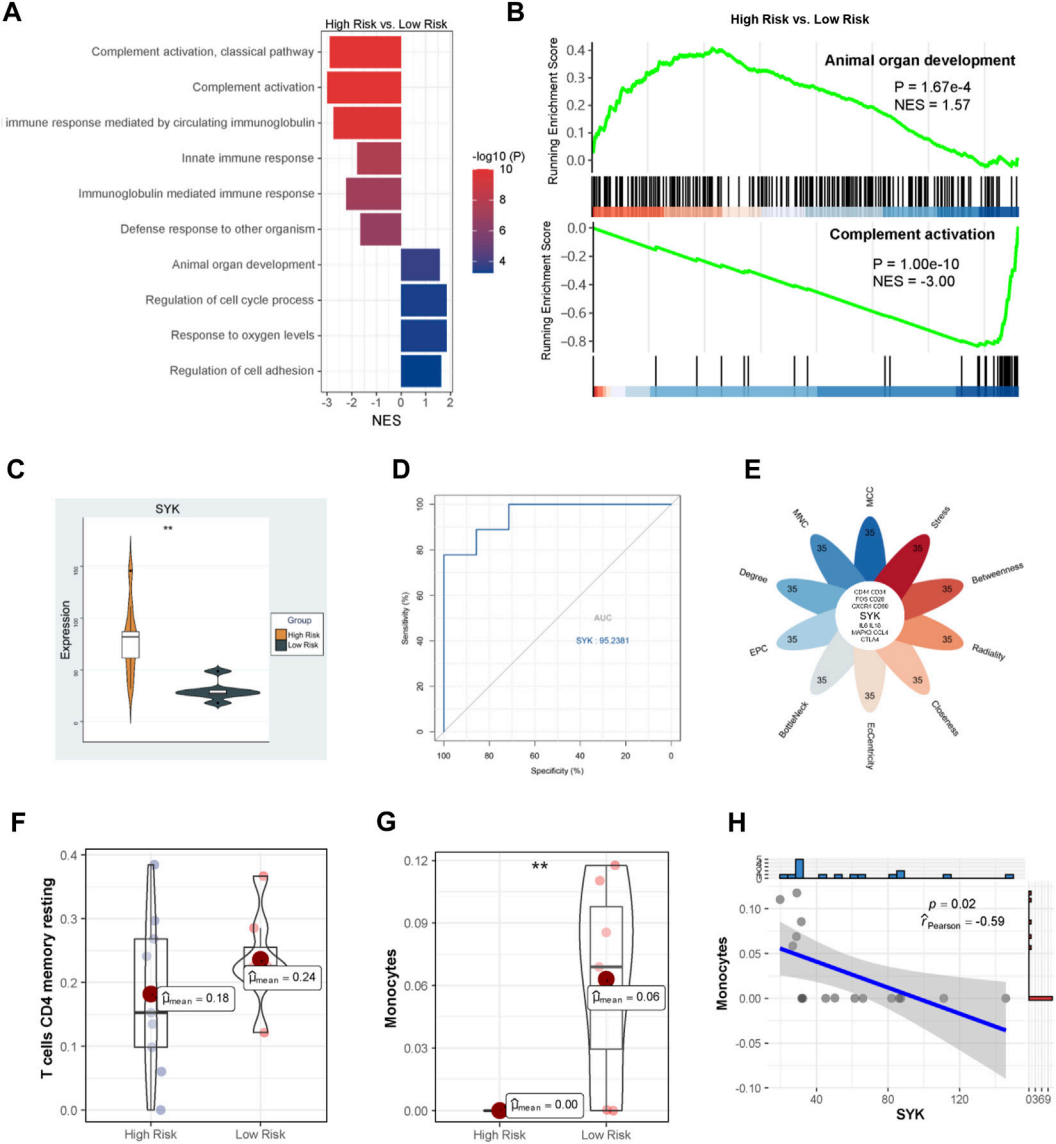
Molecular mechanism of UDCA response. **(A)** GO analysis via GSEA; **(B)** The most significantly altered GO terms; **(C)** SYK expression between the high-risk and low-risk PBC patients; **(D)** The ROC curve analysis of SYK; **(E)** Identification of hub genes; Infiltration analysis of resting memory CD4 T cells **(F)** and monocytes **(G)**; **(H)** Correlation between SYK expression and monocyte proportion.

UDCA is an important therapeutic agent for cholestatic liver injury; however, its efficacy is limited as mentioned above. For example, almost 40% of PBC patients have an insufficient response to UDCA monotherapy, are considered high-risk, and usually have a poor prognosis. We further explored which cholestasis-related modulators were associated with the therapeutic effect of UDCA using GSE79850. GSEA indicated that BPs like complement activation and innate immune response were inhibited, whereas animal organ development, cell cycle processes, response to oxygen levels, and cell adhesion were activated in the high-risk group (Figure 7A). The most significant downregulated and upregulated BPs are also shown in Figure 7B. Among the eight hub genes identified above, only SYK was found to be dramatically increased in the high-risk group compared with the low-risk group (*p* < 0.01, Figure 7C). Furthermore, SYK was considered a potential marker of poor UDCA response with a 0.95 AUC-ROC (Figure 7D). In addition, as shown in Figure 7E, SYK is also one of the key genes in the high-risk *vs*. low-risk comparison according to multiple algorithms, suggesting that SYK may play a dominant role in the mechanism of the UDCA response. We then evaluated the infiltrations of monocytes and resting memory CD4 T cells, which were considered cholestasis-related, using CIBERSORT algorithm in GSE79850. Results showed that the proportion of resting memory CD4 T cells remained compatible between the two groups (Figure 7F), whereas reduced monocytes were observed in the high-risk group (*p* < 0.05, Figure 7G). Furthermore, the expression of SYK had a significant correlation with monocytes (Figure 6H). We supposed that impaired SYK and associated monocyte infiltration in the cholestatic liver also participated in the response to UDCA therapy.

### SYK and related function variations

**FIGURE 8:**
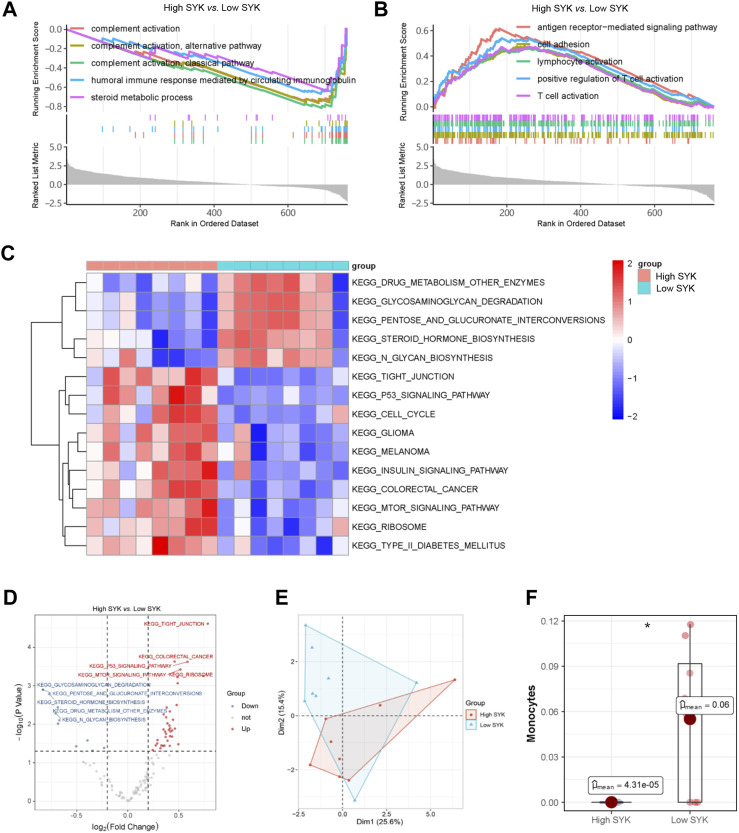
SYK and potential mode of action. Upregulated **(A)** and downregulated **(B)** GO terms in the high-SYK group via GSEA; **(C, D)** KEGG pathway analysis via GSVA; **(E)** Immune cell composition via PCA; **(F)** Monocyte proportion between the high-SYK and low-SYK groups.

In order to figure out how SYK affected UDCA response in PBC, we divided patients in GSE79850 into the high-SYK group and the low-SYK group according to the median expression of SYK. GSEA suggested that complement activation and humoral immune response mediated by circulating immunoglobulin were downregulated (Figure 8A), whereas BPs like antigen receptor-mediated signaling pathway and cell adhesion were significantly upregulated (Figure 8B) in the high-SYK group. Thus, we suspected that SYK may be involved in the UDCA response through regulating the immune response. Additionally, GSVA was also performed to identify key pathways. The results showed pathways involving drug metabolism, glycosaminoglycan degradation, and steroid hormone biosynthesis were downregulated in the high-SYK group. In contrast, pathways that were promoting tumorigeneses, like those related to glioma, melanoma, and colorectal cancer, and the p53 signaling pathway, were dramatically upregulated in the high-SYK group, implying that abnormally elevated SYK may be a potential tumor promoter (Figures 8C, D). Furthermore, a discrepant immune microenvironment was determined by PCA between the high-SYK and low-SYK groups (Figure 8E), and a decreased proportion of monocytes was consistently found in the high-SYK group (Figure 8F). Taken together, cholestasis-stimulated SYK may affect the efficacy of UDCA by regulating immune-related pathways and reducing monocyte infiltration.

### Identification of potential compounds for UDCA-refractory cholestasis

**FIGURE 9:**
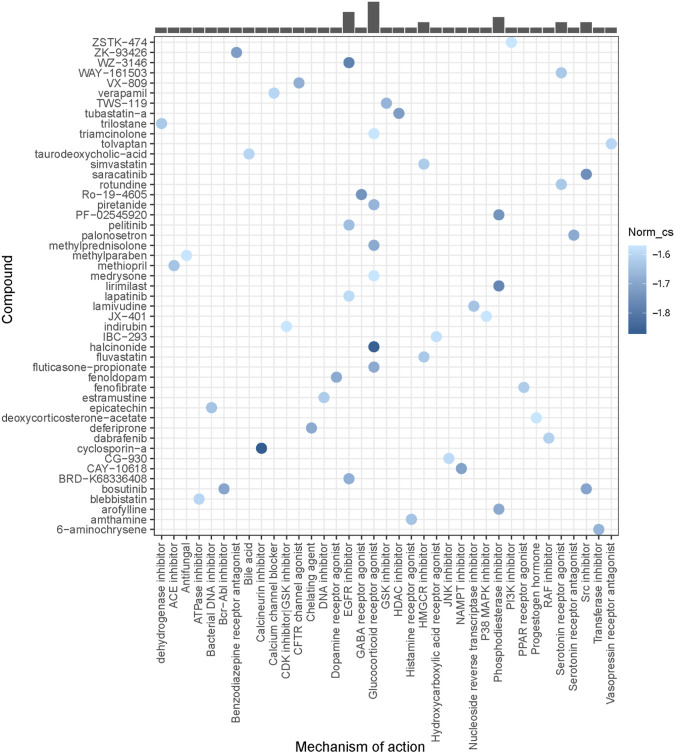
Identification of potential compounds for UDCA-refractory cholestasis.

Using DEGs between the high-risk PBC group and the low-risk PBC group, potential compounds for UDCA-refractory cholestasis were predicted by CMap analysis, and the top 50 compounds are listed in Figure 9. Among them, cyclosporin-a (a calcineurin inhibitor), halcinonide (a glucocorticoid receptor agonist), and WZ-3146 (an EGFR inhibitor) were the top 3 compounds ranked by correlation scores. Furthermore, a glucocorticoid receptor agonist was observed to be the most frequent mechanism of action. At this point, we believed that strategies aimed at glucocorticoid receptors could have a great treatment prospect for cholestasis. Interestingly, we also found an SYK inhibitor in the top 100 predicted compounds, supporting the underlying role of SYK in UDCA response.

## Discussion

Although significant progress has been made, the effective treatment of some chronic cholestatic liver diseases, such as PSC and high-risk PBC, has yet to be solved. In the present study, we first identified six immune-related DEMis and eight hub genes, as well as two immune cells, as key molecules in the process of cholestasis. Furthermore, we demonstrated that impaired SYK expression and monocyte infiltration were potentially associated with UDCA nonresponse, indicating a promising therapeutic target for cholestasis.

At first, we excavated totally six hepatic DEMis (miR-122, miR-30e, let-7c, miR-107, miR-503, and miR-192) between PBC/PSC patients and controls, and most miRNAs were confirmed in ANIT-induced or BDL-induced cholestatic mice by RT-qPCR. The effects of miR-122, miR-30e, let-7c, miR-503, and miR-192 in fibrogenesis have been extensively studied (Halasz et al., 2015; Feng et al., 2017; Devaraj et al., 2021; Wang et al., 2021; Xie et al., 2021). Besides, their associations with hepatic lipid metabolism and hepatocellular proliferation were also reported in individual studies (Wilfred et al., 2007; Sasaki et al., 2019; Sun et al., 2021). However, evidence is limited concerning cholestatic liver injury. A previous gene analysis of PSC, PBC, and biliary atresia (BA) (Luo et al., 2018) revealed that miR-122 was the only dysregulated miRNA shared by all three conditions. In addition, circulating miR-122 was identified as a biomarker for cholestatic liver diseases (Shifeng et al., 2013). These findings supported the potential role of miR-122 in cholestasis, as we found in the present study. In addition, altered expression of hepatic miR-30e and miR-192 in PBC patients was found in Padgett’s research (Padgett et al., 2009). In addition to these findings, the potential regulatory mechanisms by which these miRNAs are involved in cholestatic liver injury have yet to be figured out and necessitate deep illumination.

It is well known that immune dysregulation is an important subsequent response to cholestasis. The release of pro-inflammatory factors induced by the accumulation of bile acids promoted peripheric immune cell immigration and transformed resident immune cells into hepatocyte-damaging states. As a result, in addition to direct exposure to toxic hydrophobic bile acids, cholestatic liver injury also depended on the impaired activation of the immune system (Aller et al., 2019). In the present study, we found eight key genes involved in cholestasis, and all of them were highly immune-associated. Among them, SYK, PTPRC, TYROBP, LCP2, and CD53 are preferentially expressed on various adaptive immune cells, playing an important role in the activation of T cells and B cells (Donovan and Koretzky, 1993; Cornall et al., 2000; Dunlock et al., 2022; Iyer et al., 2022). Simultaneously, emerging evidence showed that SYK was required for monocyte adhesion (Chang et al., 2012) and was related to the phenotype switch of monocyte-derived macrophages (Chen et al., 2021), playing an indispensable role in innate immunity. TLR2 is well known to be involved in pathogen recognition and activation of innate immunity (Oliveira-Nascimento et al., 2012). Besides, RAC2 is essential for neutrophil homeostasis (Gomez et al., 2008), and LAPTM5, as a lysosomal membrane protein, has been proven to be a negative regulator of T and B cell receptor levels at the plasma membrane (Ouchida et al., 2008; Ouchida et al., 2010). Consistently, our functional analysis revealed that targeted DEMs were mostly involved in lymphocyte differentiation and activation, and regulation of the immune system. Actually, the importance of immune response in the cholangiopathies as well as cholestasis has been suggested by a previous study (Luo et al., 2018) which identified core genes shared by the three main human cholangiopathies (PSC, PBC, and BA) and showed that immune-related genes were enriched. To gain better insight into the cholestatic immune landscape, an immune infiltration analysis was performed. Results showed that increased resting memory CD4 T cells and reduced monocytes were observed in both PSC and PBC livers and were thus more likely to be involved in cholestasis. Previous studies described dysfunctional T cell response in cholestatic mice. Mechanistically, this might be attributed to bile acids disrupting intracellular calcium homeostasis, which is essential for T cell activation and results in poor hepatitis outcome (Ding et al., 2022). In addition, an increasing number of monocyte-derived macrophages were activated and recruited to cholestatic liver, which was attributed to inflammasome activation and proinflammatory cytokine secretion brought on by hydrophobic bile acids stimulation (Duwaerts et al., 2013; Gong et al., 2016). And that may explain the reduced monocytes found in this study. Notably, proportions of the two immune cells were significantly correlated with the expressions of either the identified DEMis or the hub genes, suggesting the present miRNA-mRNA regulatory network has an influence on the two immune cells, and eventually results in cholestatic liver injury. In addition, impaired Tregs and naïve B cells were observed in PSC livers compared with control livers. NK cells and macrophages were found to be altered in PBC livers compared with control livers. Besides, these findings have been explored in some experimental studies. Diminished Tregs have been described in PSC and were associated with IL2RA gene polymorphisms (Sebode et al., 2014). And B cell infiltration was considered to be related to hepatic fibrosis in *Mdr2* ^*−/−*^ mice (a classic PSC animal model) (Thapa et al., 2020). Additionally, previous studies showed that NK and macrophages are recruited to the site of liver insult and can exacerbate inflammation in PBC (Chuang et al., 2006; Shimoda et al., 2011). Although these immune cells were not the predominantly altered cells in cholestasis in the present study, their impact on cholangiopathies is unignorable and worthy of being further explored.

UDCA, as the representative agent for cholestasis, has limited efficacy in some chronic cholangiopathies, including PSC and high-risk PBC. Thus, timely identifying the potential molecular mechanisms and promoting the development of drug treatment is pressing. Since key modulators in cholestasis have been determined in the previous part of the study, we speculated that these genes may be involved in the UDCA response in patients with high-risk PBC. We chose hub genes instead of the identified miRNAs for further analysis because these miRNAs function through the targeted genes. Results indicated that elevated SYK expression was associated with high-risk PBC. Further explorations showed that complement system and monocyte infiltration were suppressed in high-risk PBC and high-SYK groups, supporting the point that SYK affected the response to UDCA through regulating immune activation. Regarding PSC, the efficacy of UDCA is uncertain and there are few studies exploring the role of SYK. Interestingly, previous studies showed that SYK was associated with the development of DSS-induced colitis (an animal model of ulcerative colitis). PSC and inflammatory bowel disease (IBD) are considered to be closely associated since most PSC patients have underlying IBD. And some common pathogenesis may be shared by the two diseases. Experimental evidence showed that SYK inhibitor significantly alleviated DSS-induced and TNBS-induced colitis by regulating TGFβ, TNFα, and IL-1β (Biagioli et al., 2017), which are also important in the course of PSC. We hypothesized that SYK might participate in the pathogenesis of PSC via some analogous pathways. Intriguingly, a SYK inhibitor was predicted to be a promising agent for UDCA-refractory cholestasis by CMap analysis. To substantiate this prediction, more experimental and clinical evidence is needed.

## Conclusion

The present study constructed a miRNA-mRNA regulatory network of cholestatic liver injury, and miR-122, miR-30e, let-7c, miR-107, miR-503, miR-192, and eight hub genes were found to regulate cholestatic liver injury by targeting immune-related pathways. Furthermore, the targeted gene SYK was also viewed as a key mediator in UDCA response.

## Data Availability

The original contributions presented in the study are included in the article/Supplementary Material, further inquiries can be directed to the corresponding author.
