# Supplementary material for: Uncovering key molecules and immune landscape in cholestatic liver injury: implications for pathogenesis and drug therapy
Source: Front Pharmacol. 2023 May 9;14:1171512. doi: 10.3389/fphar.2023.1171512 (PMC10203247; doi:10.3389/fphar.2023.1171512)
Supplement: Supplementary file 1 [file Table1.docx]

**Supplemental Table S1**Primers for RT-qPCR

| **MiRNA/mRNA** | **Primer sequence (from 5’ to 3’)** |
| --- | --- |
| mmu-miR-122 | F: CGCGTGGAGTGTGACAATGG |
| mmu-miR-30e | F: GCGCGTGTAAACATCCTTGAC |
| mmu-let-7c | F: GCGCGTGAGGTAGTAGGTTGT |
| mmu-miR-107 | F: GCGAGCAGCATTGTACAGGG |
| mmu-miR-503 | F: CGTAGCAGCGGGAACAGTT |
| mmu-miR-192 | F: GCGCGCTGACCTATGAATTG |
| U6 | F: CTCGCTTCGGCAGCACA  R: AACGCTTCACGAATTTGCGT |
| PTPRC | F: ATGGTCCTCTGAATAAAGCCCA  R: TCAGCACTATTGGTAGGCTCC |
| TYROBP | F: GAGTGACACTTTCCCAAGATGC  R: CCTTGACCTCGGGAGACCA |
| LCP2 | F: AGAGGACTTCCTGTCTGTATCAG  R: TGGACCCTCGATTCTTTCCATC |
| RAC2 | F: GACAGTAAGCCGGTGAACCTG  R: CTGACTAGCGAGAAGCAGATG |
| SYK | F: CTACCTGCTACGCCAGAGC  R: GCCATTAAGTTCCCTCTCGATG |
| TLR2 | F: GCAAACGCTGTTCTGCTCAG  R: AGGCGTCTCCCTCTATTGTATT |
| CD53 | F: AGCAGCCTGAAATTGCTGAAA  R: GGACCAGGAAATAGATGCCAAAG |
| LAPTM5 | F: GATGCCGTACCTCAGGATGG  R: CTCCCGGTTCTTGACCACG |
| GAPDH | F: AGGTCGGTGTGAACGGATTTG  R: TGTAGACCATGTAGTTGAGGTCA |

F：forward sequence; R: reverse sequence.
