# Supplementary figures and images for: Uncovering key molecules and immune landscape in cholestatic liver injury: implications for pathogenesis and drug therapy
Source: Front Pharmacol. 2023 May 9;14:1171512. doi: 10.3389/fphar.2023.1171512 (PMC10203247; doi:10.3389/fphar.2023.1171512)

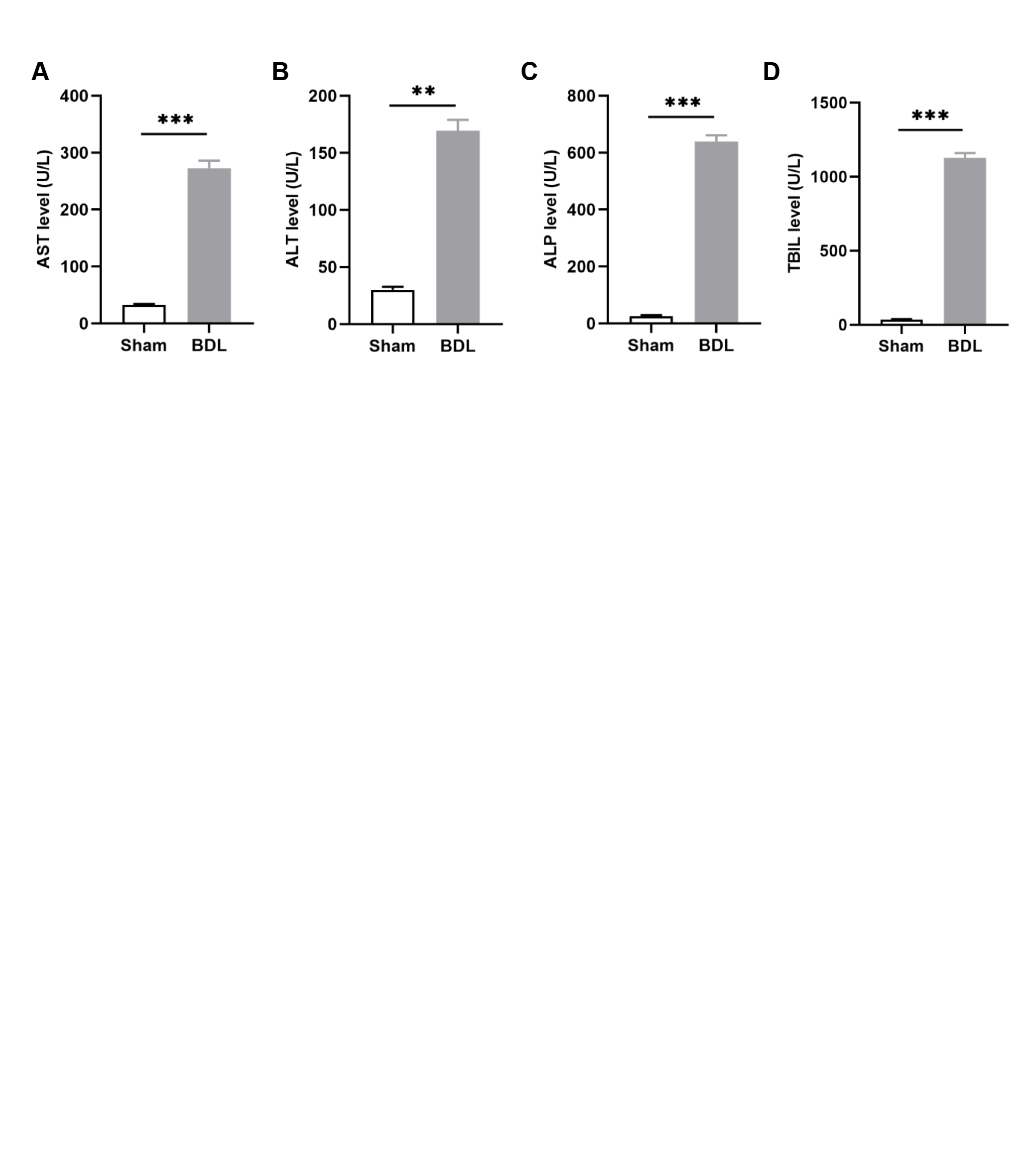

Supplement: Supplementary file 2 [file Image2.TIF]

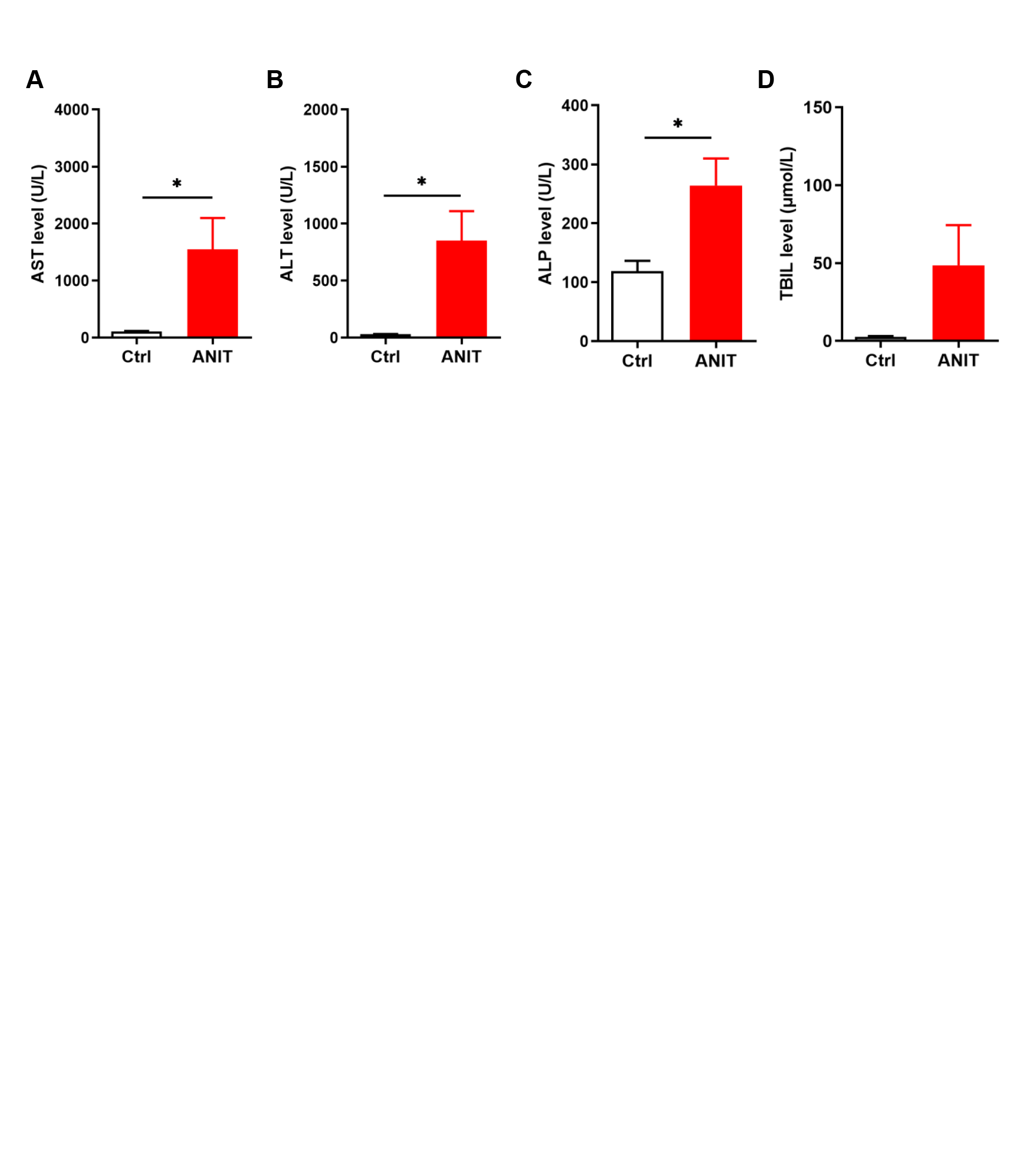

Supplement: Supplementary file 3 [file Image1.TIF]
