# Supplementary material for: Uncovering key molecules and immune landscape in cholestatic liver injury: implications for pathogenesis and drug therapy
Source: Front Pharmacol. 2023 May 9;14:1171512. doi: 10.3389/fphar.2023.1171512 (PMC10203247; doi:10.3389/fphar.2023.1171512)
Supplement: Supplementary file 4 [file DataSheet1.DOCX]

**Supplementary figure legend**

**Supplementary figure S1** Serum biochemical indexes of ANIT-induced cholestatic liver injury mice. AST, aspartate transaminase; ALT, alanine transaminase; ALP, alkaline phosphatase; TBIL, total bilirubin. *P < 0.05, **P < 0.01, ***P < 0.001.

**Supplementary figure S1** Serum biochemical indexes of BDL-induced cholestatic liver injury mice. AST, aspartate transaminase; ALT, alanine transaminase; ALP, alkaline phosphatase; TBIL, total bilirubin. *P < 0.05, **P < 0.01, ***P < 0.001.
